# Diagnosis and Treatment of a Neck Node Swelling Suspicious for a Malignancy: An Algorithmic Approach

**DOI:** 10.1155/2010/581540

**Published:** 2010-05-30

**Authors:** A. J. M. Balm, M. L. F. van Velthuysen, F. J. P. Hoebers, W. V. Vogel, M. W. M. van den Brekel

**Affiliations:** ^1^Department of Head and Neck Oncology and Surgery, The Netherlands Cancer Institute, Antoni van Leeuwenhoek Hospital, 1066 CX Amsterdam, The Netherlands; ^2^Department of Otolaryngology, Academic Medical Center, 1066 CX Amsterdam, The Netherlands; ^3^Department of Pathology, The Netherlands Cancer Institute, Antoni van Leeuwenhoek Hospital, 1066 CX Amsterdam, The Netherlands; ^4^Department of Radiotherapy, The Netherlands Cancer Institute, Antoni van Leeuwenhoek Hospital, 1066 CX Amsterdam, The Netherlands; ^5^Department of Nuclear Medicine, The Netherlands Cancer Institute, Antoni van Leeuwenhoek Hospital, 1066 CX Amsterdam, The Netherlands

## Abstract

*Aim*. To present an up-to-date algorithm incorporating recent advances regarding its diagnosis and treatment. *Method*. A Medline/Pubmed search was performed to identify relevant studies published in English from 1990 until 2008. Only clinical studies were identified and were used as basis for the diagnostic algorithm. *Results*. The eligible literature provided only observational evidence. The vast majority of neck nodes from occult primaries (>90%) represent SCC with a high incidence among middle aged man. Smoking and alcohol abuse are important risk factors. Asiatic and North African patients with neck node metastases are at risk of harbouring an occult nasopharyngeal carcinoma. The remainder are adenocarcinoma, undifferentiated carcinoma, melanoma, thyroid carcinoma and Merkel cell carcinoma. Fine needle aspiration cytology (FNAC) reaches sensitivity and specificity percentages of 81% and 100%, respectively and plays an important role as the second diagnostic step after routine ENT mirror and/or endoscopic examination. FDG-PET/CT has proven to be helpful in identifying occult primary carcinomas of the head and neck, especially when applied as a guiding tool prior to panendoscopy, and may induce treatment related clinical decisions in up to 60% of cases. *Conclusion*. Although reports on the diagnostic process offer mainly descriptive studies, current information seems sufficient to formulate a diagnostic algorithm to contribute to a more systematic diagnostic approach preventing unnecessary steps.

## 1. Introduction

Patients with a suspicious lump in the neck are regularly seen. The overwhelming amount of possible diseases linked to a swelling in the neck makes it of utmost importance to follow a strict protocol for appropriate diagnosis making. If not, this might lead to a considerable diagnostic delay [1].

Neck node metastases from an unknown primary site (UPS) are part of the “Cancer of Unknown Primary” origin, where the primary tumor may remain unknown for a patient's lifetime despite thorough diagnostic work-up [2]. This clinical entity may develop by complete involution of the primary or by a genetic influence, favoring metastatic growth over primary tumor growth [3]. Although approximately one third of metastases from UPS are found in lymph nodes [4], the incidence of neck nodes from UPS makes up only 1.7%–5.5% of all head and neck carcinomas in large series [1, 5, 6]. Over 90% of neck metastases comprises squamous cell carcinoma (SCC) [7] whereas adenocarcinoma, undifferentiated carcinoma, and other malignancies (e.g., thyroid carcinoma, melanoma) are less common in the Western world. Undifferentiated carcinomas are more often seen in countries with a high prevalence of nasopharyngeal carcinomas.

## 2. Method

The Medline/Pubmed database was searched by using neck node, cervical adenopathy, unknown primary, occult primary, and metastasis as search terms to identify relevant studies published in English from 1990 until 2008. Out of 226 selected papers 34 relevant papers were selected after reviewing the abstracts by two experienced head and neck surgeons, a nuclear physician and a radiotherapist. Only clinical descriptive studies were identified. These and two Dutch publications [1, 8] were used as basis for a diagnostic algorithm. Recommendations made in this paper reach a level IV evidence (expert opinion).

### 2.1. Initial Diagnostic Work-Up of a Suspicious Lump in the Neck

A neoplastic nature should be considered firstly in patients beyond the age of 40 years, particularly those with a history of alcohol abuse and heavy smoking. Racial traits are also important: masses in the upper neck levels of Asiatic, North African, and Indian patients are often related to occult carcinoma in the nasopharynx [10] and oropharynx/oral cavity [11], respectively.

**Figure 1:**
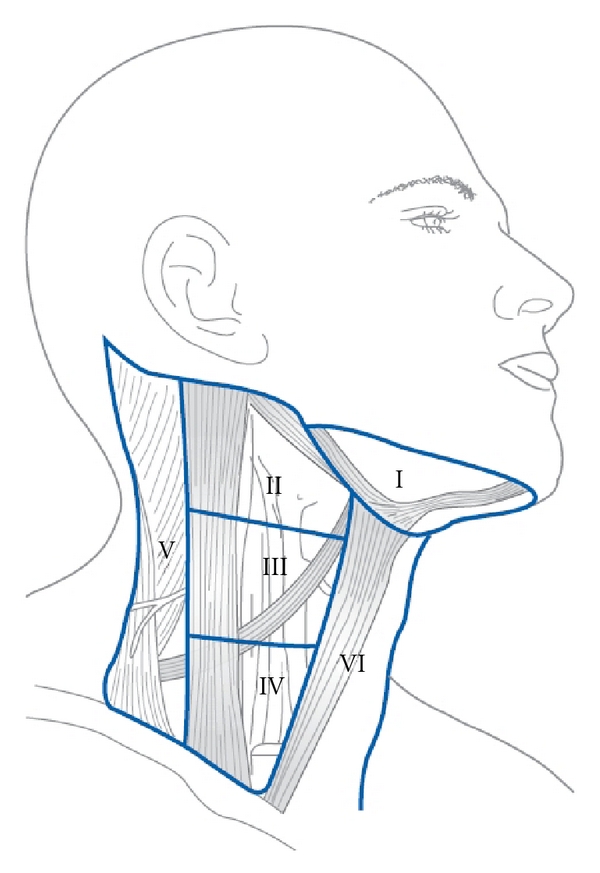
The 6 sublevels of the neck according to Robbins et al. [9] (Figure printed with permission of the publisher Bohn Stafleu van Loghum, Houten, the Netherlands).

Node metastases can be found in every neck level (Figure 1), with metastases from UPS most frequently found in level II [1, 5, 12, 13]. Neck nodes from UPS present bilaterally in 10% of cases [12, 14]. In general, nodes in levels I–III are attributed to a presumable primary SCC located in the mucosa of the upper aerodigestive tract [15–17], whereas nodes in levels IV and Vb more often arise from proximal esophageal and thyroid carcinomas, but can also originate from distant organs in the body, often containing adeno- or large cell undifferentiated carcinoma (LCUC). Lymph nodes with adenocarcinoma are frequently accompanied by multiple metastatic sites, such as lung, liver, and bones as part of the CUP syndrome [18, 19]. Lymph nodes in level IIb and Va are more typical for nasopharyngeal cancer. Nodes in the parotid area originate most often from skin cancer and should be distinguished from primary parotid tumors and level I metastases from primary submaxillary gland carcinomas. Melanoma containing nodes may occur in every level of the neck, often involving superficial, nuchal, level V, and parotid lymph nodes [20].

When a suspicious node has been found, accurate examination of the upper aerodigestive tract mucosa by mirror examination and/or fiber-optic or rigid endoscopy is required, as well as (bimanual) palpation of the oropharynx and mouth. If this results in the detection of a primary carcinoma, further specific diagnostic measures can be taken. If no primary tumor is detected, the next diagnostic step is the fine needle aspiration cytology (FNAC) of the node by an experienced cytologist or surgeon. If the lesion is more difficult to approach or cytology is nondiagnostic, ultrasound-guided fine needle aspiration cytology (USFNAC) has to be performed.

### 2.2. Evaluation of Histopathological Characteristics

Neck metastases present mostly as firm, solid masses, but a distinct subset of metastatic nodes present as cystic masses frequently related to thyroid carcinoma followed by SCC and malignant melanoma [21]. Goldenberg et al. [22] observed that certain SCCs of the tonsil are more likely to produce cystic metastases. Today, it is concluded that so-called “Branchiogenic carcinomas” represent cystic alteration in neck metastasis in stead of a branchiogeneic carcinoma. Recently, a subgroup of patients with cystic lymph node masses related to SCC, that often lack the presence of the classical risk factors for SCC, has been identified and related to HPV-associated SCC [23]. The presence of HPV can be detected in FNAC material [24] and directs the search for a primary cancer arising in the oropharynx. However, cystic masses will often lead to a negative misleading FNAC finding, indicating repeated ultrasound-guided FNAC from solid parts in the cysts [8, 25]. In general, high sensitivity and specificity percentages of 81% and 100%, respectively, are reported for FNAC allowing the clinician to be confident of malignancy in a clinically suspicious lesion [26–29], but lower specificity percentages (57%) have also been found [30]. Cheng and Dorman [26] conclude that diagnostic accuracy improves with experience and good communication between cytopathologist and clinician. Only repetitive negative or nondiagnostic FNACs are an indication for an incisional, excisional, or (image-guided) true-cut biopsy. Although a direct effect of a neck node biospsy on tumor recurrence has not been demonstrated [31], we still do not advise to perform an incisional biopsy because of the adverse effect on subsequent surgery of the neck by scar formation. In case of a lymphoid proliferation, Fluorescence-Activated Cell Sorter (FACS) analysis of the aspirate collected in RPMI medium is helpful in distinguishing reactive lymph nodes from NHL with monoclonal lymphoid cell proliferation. This method is less invasive than fresh lymph node biopsies and with similar result. However, for definitive NHL classification a complete (fresh) lymph node excision is needed

### 2.3. FNAC: Squamous Cell Carcinoma

Cytological diagnosis of SCC and negative routine ENT and skin examination results, require accurate panendoscopy under general anaesthesia of the mucosal lining of the upper aerodigestive tract. It can be advised to let endoscopy be preceded by a dedicated imaging process consisting of either MRI (3-4 mm slice thickness) or FDG-PET(/CT) to improve the yield of the procedure [32–35]. Positive predictive values range from 88% to 62% for FDG-PET and conventional imaging (CT and-/or MRI), respectively [36]. Early investigations with FDG-PET have shown equivocal or disappointing results for the detection of occult primary tumors [37–39]. Ongoing technical improvements in PET image resolution and sensitivity and integration with CT [40] have resulted in better clinical value. In recent publications, FDG-PET was shown to detect occult primary cancer sites in about 30% of cases after negative clinical and radiological work-up [41, 42], and including panendoscopy [43]. With implementation of the positive predictive value, FDG-PET seems only useful in approximately 25% of the patients. The optimal place for FDG-PET in the diagnostic algorithm seems to be prior to panendoscopy, to avoid false-positive results due to prior biopsies [41], and to improve the yield of panendoscopy by guiding biopsy based on the PET results [43]. In addition, FDG-PET as a wholebody procedure may also improve staging by demonstrating occult regional and metastatic disease [39, 41]. In total, adding FDG-PET may induce-treatment related clinical decisions in up to 60% of cases [43]. The value of a negative FDG-PET scan has also been recognised. Most patients with no evidence of a primary on FDG-PET will never develop a clinically recognisable primary tumor, especially those who also have negative findings on panendoscopy and MRI [42, 44]. False-negative and false-positive FDG-PET results do occur, especially in the tonsillar regions, where physiological inflammation may obscure metabolic tumor activity [45]. Therefore, FDG-PET(/CT) has a place early in the diagnostic algorithm for detection of an unknown primary in head and neck, but cannot preclude the need for panendoscopy with biopsy to detect occult primary tumors [42]. Whilst MRI and/or PET/CT is the investigation of choice prior to panendoscopy, CT scanning should be acknowledged as an acceptable alternative for those clinicians who do not have ready access to these imaging modalities.

During the process of careful mucosal examination one should keep in mind that in the Western world the base of tongue and tonsillar area are the predilection subsites for harboring an occult superficially growing primary SCC [16], whereas in Asia it is the nasopharynx. Suspicious findings on clinical examination or imaging studies can direct biopsies for pathological confirmation of a primary carcinoma. In case of a normal macroscopic appearance of the whole mucosa, it is recommended to do an additional tonsillectomy [46], since approximately one quarter of primary tumors are detected at this site [34, 47–49]. For those patients with both negative endoscopic and imaging findings, it can be questioned whether ipsilateral tonsillectomy and blinded biopsies from the base of tongue, nasopharynx, and piriform sinus are to be recommended [42, 44]. It is not clear from the literature, whether bilateral tonsillectomy has been established as the standard procedure in cancer of unknown primary origin [50]. The rate of contralateral spread of metastatic cancer from occult tonsil lesions appears to approach 10% [51]. For this reason, bilateral tonsillectomy can be recommended without losing time for adequate treatment.

**Figure 2:**
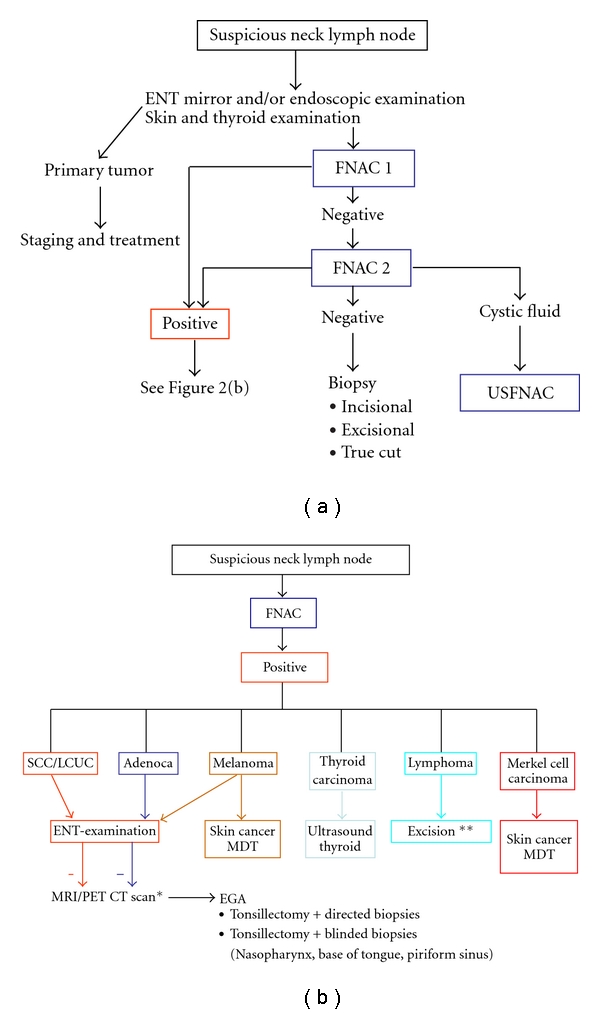
(a) Algorithm of a diagnostic procedure for a suspicious lymph node in the neck, emphasizing the pivotal role of (Ultrasound) Fine Needle Aspiration Cytology [(US)FNAC]. (b) Algorithm of a diagnostic procedure for a suspicious lymph node in the neck with emphasis on further steps to be taken after positive FNAC results. EGA: examination under general anaesthesia; LCUC: large cell undifferentiated carcinoma; MDT: multidisciplinary team; SCC: squamous cell carcinoma; *CT scan can serve as an acceptable alternative; **Excision indicated if FACS (Fluorescence activated cell sorting by Flow Cytometry) reveals monoclonal lymphoid proliferation.

With the above described algorithmic approach (Figures 2(a) and 2(b)), only 2% of head and neck malignancies will finally be classified as originating from unknown primary site in the head and neck region [1].

The rising incidence of cutaneous SCC worldwide makes it increasingly likely that clinicians may also encounter regional nodal metastases of this type of skin cancer in the parotid gland and the upper levels of the neck [52]. Therefore, high risk areas such as ear and scalp should also be examined thoroughly. If neck nodes are found in levels Va and Vb, examination of the skin of the neck and torso should be performed as well. Although rare, Merkel cell carcinoma is another cutaneous aggressive malignancy of which half of the cases presents primarily in the head and neck with a high propensity for neck node metastases [53].

The absence of an exact location of the primary creates an enormous dilemma, particularly with respect to whether all potential primary tumor sites need to be treated with radiotherapy [34, 47, 54]. In theory, radiation therapy could be given to the involved ipsilateral neck only, to candidate primary sites and involved neck, or to the bilateral neck and potential primary sites. All published studies on this topic are of retrospective nature. However, some of these papers suggest that inclusion of extensive radiation to candidate primary sites and bilateral neck results in less locoregional failures as compared to ipsilateral neck radiation only [5, 41, 55]. Summarizing the available retrospective literature data, Nieder et al. [34] concluded that patients receiving nodal resection and bilateral neck irradiation, including the potential primary mucosal sites, appeared to have better locoregional control than neck surgery with ipsilateral radiation or radiotherapy alone. On the other hand, some retrospective single-institution studies reported that radiation to the ipsilateral neck only was not associated with decreased survival or higher emergence rates of mucosal primaries [5, 56–58]. A confounding factor in these series may be the fact that in reported unilateral irradiation to the neck alone a considerable portion of the (ipsilateral) mucosal sites might have received radiation due to the use of nonconformal radiation techniques in the past.

There is no direct comparative data on the use of ipsilateral mucosal irradiation (e.g., to the tonsillar fossa or the lateralized base of tongue) versus extensive bilateral mucosal irradiation with regard to the prevention of occurrence of primary tumors. However, some of the previously quoted [5, 56–58] ipsilateral neck radiation series (that will have included ipsilateral mucosal irradiation too) did not report increased rates of emergence of primary tumors compared to extensive radiation.

One obvious concern of extensive radiation is the development of normal tissue toxicity, including long-term xerostomia which may significantly decrease quality of life [59]. Modern radiation therapy techniques like IMRT may overcome some of these issues and result in sparing (one of) the salivary glands. One should weigh carefully the different treatment modalities like neck surgery alone [(selective) neck dissection], comprehensive irradiation of bilateral cervical nodes versus radiation to the ipsilateral neck along with putative primary sites, nodal excision or selective neck dissection followed by radiotherapy or primary chemoradiation with salvage neck dissection in reserve.

Considering toxicity, a patient-tailored-individualized treatment approach could be applied consisting of ipsilateral radiation only in case of favorable neck disease after neck dissection (e.g., pN1 without extranodal extension). In cases with bilateral nodal metastases, extensive unilateral involvement with regard to number and levels of nodal metastases, or extranodal extension, comprehensive radiation should be considered. Unfortunately, a randomized trial by EORTC and RTOG to investigate whether or not comprehensive radiation to bilateral neck and potential candidate mucosal sites would lead to improved results compared to ipsilateral neck radiation only failed due to poor accrual.

### 2.4. Adenocarcinoma

Adenocarcinoma containing neck nodes from UPS are exceptional [7], theoretically occurring in any neck level. If detected in levels I–III, they raise a high suspicion for a metastasis from a salivary gland carcinoma, including also the small submucosal salivary glands. Metastases from a thyroid gland carcinoma are mainly found in levels II–VI. Metastases located in level IV and VB (supraclavicular) are exceptional and, if not from thyroid origin, suspicious for the existence of a primary carcinoma below the clavicles (lung, mammary gland, prostate, and gastro intestinal tract).

CT scans of the lungs and abdomen, mammography, ultrasound of the thyroid, and urological examination, including determination of PSA serum values in males and CEA in all patients, should be used for clinical work-up. PET-CT scan can replace conventional CT for the identification of the primary tumor with a reported detection rate of 57% [60]. For neck node metastases in levels I–III, we also advise a thorough fiber endoscopic ENT examination, including ultrasound of the parotid glands.

In some cases, the cytopathologist can do several immunohistochemical analyses with the embedded aspirate to help distinguish different adenocarcinomas [61–63].

In the majority of patients presenting with an adenocarcinoma in the neck, it is the first sign of disseminated disease. Nevertheless, in isolated neck nodes a prolonged survival of median 25 months can be achieved by a selective neck dissection followed by radiotherapy [19]. See for algorithm Figures 2(a) and 2(b).

### 2.5. Large Cell Undifferentiated Carcinoma (LCUC)

Isolated metastases from LCUC may either be derived from an occult primary SCC of the head and neck or an adenocarcinoma. Combination of FNAC and immunohistochemistry and interpretation by an experienced head and neck pathologist is of utmost importance. For a definitive distinction between adenocarcinoma and LCUC recognition of growth patterns and mucin stains remains a crucial part of the diagnostic process [64]. In those cases where the final cytological diagnosis of an LCUC remains, algorithmic pathways for the search of an occult primary of both adenocarcinoma and SCC should be implemented. The possibility of a primary salivary gland carcinoma should be taken into account. If no primary has been found, a LCUC is part of a classical CUP syndrome, with a very poor prognosis. Nevertheless also for these cases a selective neck dissection followed by radiotherapy might lead to good palliation or even prolonged survival [19]. See for algorithm Figures 2(a) and 2(b).

### 2.6. Melanoma

A metastasis in the neck or parotid region from an unknown primary melanoma is a rare event. Out of a total of 300 patients with head and neck melanoma 17 (5.7%) presented in this way [20]. The work-up of a patient with a metastatic melanoma from unknown origin requires an active participation of the dermatologist in the search for the primary lesion in the skin of the face and scalp. It is also advisable to perform routine ENT examination to search for an occult melanoma of the head and neck mucosa [65]. Additional imaging seems of limited value in detecting the primary lesion [7], but can be considered to exclude disseminated disease, especially in patients presenting with positive lymph nodes. In a systematic review by Krug et al. [66] wholebody imaging with FDG-PET(/CT) of patients with AJCC stage III+ revealed distant metastases with sensitivity 83% and specificity 85%, with impact on treatment in 33%. In patients with no distant metastases, modified radical neck dissection is still the mainstay of treatment. Posterolateral neck dissection, removing levels II–V, is indicated if the metastasis is located in level V. This dissection should be extended to the postauricular and occipital lymph node basins. Postoperative radiotherapy may improve locoregional control for patients with bad prognostic signs, that is, multiple nodes and/or extracapsular spread [67]. See for algorithm Figures 2(a) and 2(b).

### 2.7. Thyroid Carcinoma

Well-differentiated thyroid carcinomas as well as medullary and undifferentiated thyroid carcinomas have a very high propensity to spread to the cervical lymphatics [68–70]. In fact, if the diagnosis of thyroid carcinoma is confirmed by cytology or open biopsy, it is no longer considered as metastasis of unknown primary origin. In general, metastases in levels III, IV, and VI should raise suspicion of a primary thyroid malignancy. Even in younger females, and especially in patients with familial traits (MEN1-2) or previous irradiation to the neck these thyroid malignancies can occur. Apart from a lump in the thyroid gland at either palpation or US, the cytology plays a pivotal role in the diagnosis. Well-differentiated thyroid carcinomas often have a quite characteristic appearance. IHC using antibodies to thyroglobulin, TTF-1, or calcitonin can give further direction to the differential diagnosis of this adenocarcinoma. In case of doubt, serum CEA and calcitonin should be obtained to exclude a medullary carcinoma. Undifferentiated thyroid carcinomas are less easily classified cytologically. In these patients, the clinical picture of a fast growing mass in the thyroid area in elderly patients, combined with a large cell undifferentiated cytology most often leads to the diagnosis. Lymph node metastases warrant selective modified neck dissections with sparing of internal jugular vein, accessory nerve, and sternocleidomastoid muscle, followed by ablation of functional thyroid remnants with radioactive iodine.

## 3. Conclusion

Neck node containing metastasis of carcinoma from unknown primary origin represents squamous cell carcinoma in the majority of cases among a wide variety of histologies. An algorithmic approach with careful examination of the head and neck and a pivotal role for fine needle aspiration may contribute to a more systematic diagnostic approach preventing unnecessary steps and allow for site-specific therapy. See for algorithm Figures 2(a) and 2(b).
